# Insulin resistance as estimated by the homeostatic method at diagnosis of gestational diabetes: estimation of disease severity and therapeutic needs in a population-based study

**DOI:** 10.1186/1472-6823-13-21

**Published:** 2013-07-02

**Authors:** Alina Sokup, Barbara Ruszkowska-Ciastek, Krzysztof Góralczyk, Małgorzata Walentowicz, Marek Szymański, Danuta Rość

**Affiliations:** 1 Department of Gastroenterology, Angiology and Internal Diseases, Nicolaus Copernicus University, Dr. J. Biziel University Hospital, Bydgoszcz, Poland; 2 Department of Endocrinology, Dr. J. Biziel University Hospital, Bydgoszcz, Poland; 3 Department of Pathophysiology, Nicolaus Copernicus University, Dr. A. Jurasz University Hospital, Bydgoszcz, Poland; 4 Department of Obstetrics and Gynecology, Nicolaus Copernicus University, Dr. J. Biziel University Hospital, Bydgoszcz, Poland

**Keywords:** Gestational Diabetes, Pathophysiology, Gestational Diabetes, Treatment, Gestational Diabetes, Insulin Resistance, Gestational Diabetes, HOMA

## Abstract

**Background:**

Chronic insulin resistance, exacerbated in the course of pregnancy, is an important pathophysiologic mechanism of gestational diabetes mellitus (GDM). We hypothesise that the degree of insulin resistance, assessed at diagnosis of GDM, is a parameter of its pathophysiologic heterogeneity and/or severity. Thus, it offers potential to open new avenues for the personalization of therapy in affected women.

**Methods:**

1254 Polish Caucasian women with GDM were recruited into the study. The following parameters were assessed in the course of the study: body mass index (BMI), parity, weight gain during pregnancy, glycated haemoglobin, glucose level during an oral glucose tolerance test (OGTT), insulin, insulin resistance and insulin secretion. The severity of GDM was assessed based on insulin use and daily insulin dose during gestation. In order to evaluate insulin secretion and insulin resistance the homeostatic method was used (HOMA-B and HOMA-IR, respectively). We compared all the metabolic parameters and methods of treatment of GDM in women subdivided by quartiles of insulin resistance.

**Results:**

The HOMA-IR in the whole population ranged from 0.34 to 20.39. The BMI, fasting insulin, fasting glucose and insulin dose per day increased along with increasing quartiles (HOMA-IR > 1.29). We observed a decrease of HOMA-B in the third quartile (1.92-2.89) compared with the first quartile (0.34-1.29). Insulin treatment was associated with HOMA-IR (<1.29 vs. >2.89), OR: 3.37, fasting glucose (≤6.11 vs. >6.11 mmol/dl), OR: 2.61, age (≤30 vs. >30 y. o.), OR: 1.54, and BMI (<25 vs. ≥25 kg/m^2^), OR: 1.45. Maximum insulin dose was associated with HOMA-IR, OR: 2.00, after adjustment for family history of diabetes, and 2-h OGTT glucose.

**Conclusion:**

Insulin resistance assessed by the HOMA index at diagnosis is associated with the severity and pathophysiological heterogeneity of GDM. A HOMA-IR >1.29 points to the major role of insulin resistance, indicating the need for a treatment aimed at improving tissue sensitivity to insulin. A HOMA-IR 1.29-2.89 suggests reduced insulin secretion, which is an indication for the introduction of insulin therapy. A HOMA-IR >2.89 indicates insufficient compensation for insulin resistance, which suggests the need for a treatment aimed at improving susceptibility of tissues to insulin combined with insulin therapy.

## Background

Gestational diabetes (GDM), defined as various degrees of glucose intolerance diagnosed or detected for the first time during pregnancy, is the most common metabolic complication of pregnancy. Diagnosis of GDM is associated with an increased risk of complications for the mother and child, both during pregnancy and many years postpartum [1]. This risk can be reduced by appropriate dietary means and an increase of physical activity, possibly in combination with administration of insulin or oral drugs. Despite numerous studies, the pathophysiology of GDM has not been elucidated. It is known that GDM is a heterogeneous pathophysiological state, in which the main mechanism is possibly the dysfunction of pancreatic beta cells, manifesting itself in the course of increasing insulin resistance during pregnancy [2].

Therefore, it is of great importance to search for parameters helpful in individualisation, and thus optimising GDM treatment, as well as in preventing GDM complications. The medical literature offers no studies describing strategies for GDM treatment based on pathophysiological criteria. Moreover, there is no criterion of adding pharmacological therapy to diet other than glucose level measurement [3].

Insulin resistance is an important pathogenic mechanism, which precedes GDM occurrence [4-6]. It is probably chronic in nature [4,5,7] and is associated with an increased risk to the mother and child [4]. Recent studies indicate a direct [8] and indirect [9] relationship between the insulin resistance indices in the first trimester of pregnancy and the risk of GDM development, as well as risk of macrosomy.

The HOMA-IR index is based on a single measurement of glucose and insulin in the blood [10]. It is commonly used as a parameter of the severity of insulin resistance and it strongly correlates with direct assessment of insulin resistance using the euglycemic clamp technique [11].

The aim of the current study was to evaluate the relationship of the degree of insulin resistance, assessed using the HOMA-IR index at diagnosis of GDM, with selected clinical, anthropometric, and biochemical parameters, as well as with pancreatic beta cell function, in order to consider the potential use of this index for personalized therapy. In particular, we considered the following aspects: 1* does the degree of insulin resistance assessed by the HOMA index at diagnosis of GDM differentiate the studied population pathophysiologically and clinically, 2* is the parameter associated with the treatment method, and thus can it be used as a severity marker of GDM.

## Material and methods

We carried out a prospective study in which we assessed selected anthropometric, clinical, and pathophysiological parameters, as well as the method of treatment used for gestational diabetes mellitus (GDM) in 1254 women who have already been diagnosed with GDM in the actual pregnancy. 184 women (15%) had a previous history of GDM. The criteria for inclusion in the study were an age range between18 to 35 and pregnancy resulting from spontaneous conception. Exclusion criteria were chronic diseases, acute infection at diagnosis or in the course of the pregnancy, and the receiving of drugs affecting the carbohydrate metabolism. The characteristics of the studied population are presented in Table 1.

**Table 1:** Clinical characteristics of the participants

**Characteristic**	**Values**
N	1254 patients
Age (years)	29.93 ± 5.23
Prepregnancy BMI (kg/m^2^)	28.48 ± 4.46
Gestational age at the diagnosis of GDM (week)	23.92 ± 4.91
Positive family history of diabetes	445 (35)
Number of pregnancies	2.02 ± 1.28
Diet therapy	935 (75)
Diet + insulin therapy	319 (25)

Values of parameters are presented as mean ± SD or count (percentage).

The study was conducted at the Intensive Diabetologic and Obstetric Care Unit, Nicolaus Copernicus University Hospital in Bydgoszcz, between 2003 and 2007. The project was approved by the University Local Research Ethics Committee and informed consent was obtained from all the women.

### Method of diagnosis and treatment of gestational diabetes mellitus

All the pregnant women with no previously diagnosed diabetes were offered screening for gestational diabetes mellitus (GDM) with a 1 h 50 g oral glucose challenge test (GCT) between 24 and 28 weeks of pregnancy. If fasting plasma glucose evaluated at early prenatal visit was equal or above 5.5 mmol/l (threshold ≥100 mg/dl) the GCT test was performed by the gynecologist as soon as possible accordingly to the Polish Diabetes Society recommendations. A value equal to or above 7.7 mmol/dl (threshold ≥140 mg/dl) identified women who underwent a further 2-h 75-g oral glucose tolerance test (OGTT-Oral Glucose Tolerance Test). Diagnosis of GDM was in accordance with World Health Organization criteria [12] modified by the Polish Diabetes Society. GDM was diagnosed when the fasting plasma glucose was equal to or above 5.5 mmol/l (threshold ≥100 mg/dl) in two distinct tests, or when the plasma glucose was equal to or above 7.7 mmol/l (threshold ≥140 mg/dl) two hours after a 75 g oral glucose challenge test.

All the women who were diagnosed with GDM were advised about diet (23–25 kcal/kg pregravid weight for obese, 30–34 kcal/kg pregravid weight for normal weight) and physical activity (walking two–three times daily, half an hour to one hour per day). Self-monitoring of blood glucose levels was performed 4 times/day, fasting and 1 h postprandial. Insulin was started for persistent fasting blood glucose values ≥5.0 mmol/l and/or 1 h postprandial blood glucose values ≥6.6 mmol/l after two weeks of treatment.

### Study methods

To investigate the association between the severity of insulin resistance and the following parameters: age, positive family history of diabetes in first-degree relatives, number of previous pregnancies, prepregnancy body mass index, weight gain during pregnancy at diagnosis of GDM, time of diagnosis, and GDM treatment, all studied women were divided into quartiles accordingly to HOMA-IR values.

In order to evaluate the treatment method, subjects were divided into two groups: one group treated with diet and increased physical exercise only, and women additionally treated with insulin.

We used the following criteria in order to assess the severity of GDM: introduction of insulin therapy, the minimum daily dose of insulin necessary to normalise glucose concentrations in the initial phase of treatment, and the maximum daily dose of insulin in the course of pregnancy.

To assess the independent determinants of insulin therapy in the studied population we used logistic regression analysis. In this analysis the dependent variable was the method of therapy as a categorical variable (diet vs. diet and insulin). The following factors were taken into account as an independent categorical variables within this evaluation: age (≤30 vs. >30 years), prepregnancy BMI (<25 vs. ≥25 kg/m^2^), fasting glucose at diagnosis of GDM (≤6.11 vs. >6.11 mmol/l), diabetes mellitus in first-degree relatives (yes vs. no), HOMA-IR (1st vs. 4th quartile e.g. HOMA-IR range 0,34-1,29 vs. 2,89-20,39) and weight gain at diagnosis of GDM (≤ 8 vs. > 8 kg). The parity, time of GDM diagnosis, HOMA-B index and glucose concentration in a 2-h OGTT turned out to be statistically insignificant in correlation testing and these parameters were not included in the model.

In the next step we assessed the association between minimum mean daily dose of insulin necessary to normalize glucose concentration at the beginning of therapy and HOMA-IR . The relation was assessed using logistic regression analysis in order to consider the effect of potential determinants of initial mean daily dose of insulin and insulin resistance –HOMA-IR index values. In separate logistic regression models appropriate interaction terms were introduced to assess interactions between dose of insulin as a categorical variable (1th tertile vs. 3th tertile e.g. 1–6 vs. >16-116 IU/per day) as an dependent variable: age, pre-pregnancy BMI, gestational age at diagnosis( ≤22 vs. >22 week), weight gain at diagnosis, family history of diabetes, and glucose at 2-h OGTT(8,88 vs. > 8,88 mmol/l) as independent variables.

Finally, we assessed the relation of maximal mean daily dose of insulin in the course of pregnancy and HOMA-IR index values using separate logistic regression models. The appropriate interaction terms were introduced to models interactions between maximal mean daily insulin dose as dependent categorical variable (1th tertile vs. 3th tertile, e.g. 1–22 vs. >54-240 IU/per day) and age, prepregnancy BMI, HOMA-IR index, family history of diabetes, weight gain at the diagnosis of GDM, fasting glucose, and glucose at 2-h OGTT.

The following laboratory parameters were assessed in all the women at diagnosis of GDM: glycated haemoglobin, fasting insulin concentration, plasma glucose concentration in the fasting state and 2-h after oral challenge with 75 g glucose according to WHO recommendations.

Venous blood was collected from the elbow vein and used as research material. Blood samples were collected from the patients between 7.30 am and 9.30 am after a 12-hour overnight fast. In order to determine the serum fasting glucose and insulin concentration, blood samples were collected in 4.5 ml tubes without anticoagulant, and subsequently centrifuged at 3000 *g*, 4°C for 20 minutes. The concentration of glucose was determined by the glucose oxidase method using an Olympus AU 400 analyser (reference range: 3.3-5.5 mmol/l). The concentration of insulin was measured by the immunoenzymatic method (MEIA) using AxSYM analyser (reference range: 2–25 μU/ml). The A1C in whole blood was measured by the turbidimetric method (reference values <6%). All the laboratory tests were performed at the hospital’s Department of Analytics.

The severity of insulin resistance (HOMA-IR index) and pancreatic beta cell function (HOMA-B index) were assessed on the basis of a single determination of fasting glucose and insulin concentrations, using mathematical formulas described by Mathews and colleagues [10].

### Statistical analysis

All continuous variables are presented as mean values +/− SD (standard deviation). Categorical traits are presented as a count (percentage). Quartiles of HOMA-IR were used to characterize whole studied population with respect to the degree of insulin resistance degree. Comparisons between the subgroups were performed by one-way analysis of variance (ANOVA) with post-hoc analysis to locate the differences. The p value of less than 0.05 was considered significant. Categorical variables were compared by means of the chi square test. The logistic regression method was used in assessing the relationship between the severity of insulin resistance and treatment for GDM (diet vs. diet and insulin).

Logistic regression models were used in assessing the relationship between the severity of insulin resistance and the minimum initial daily dose (low tertile vs. high tertile) of insulin or the maximum daily dose of insulin(low tertile vs. high tertile) in the course of treatment. Statistical analysis was performed using Statistica for Windows by StatSoft.

## Results

The study population of women demonstrated a relatively young age, slight obesity, and relatively early GDM diagnosis. In most women, the diet treatment was sufficient to achieve normoglycemia. Only a quarter of the respondents required the insulin therapy inclusion. The characteristics of the study population are shown in Table 1.

The characteristics of the study subjects subdivided by the HOMA-IR quartiles are reported in Table 2. The increasing degree of insulin resistance in quartiles of HOMA-IR is associated with a progressive increase in body mass index, level of insulin and fasting glucose, as well as the frequency of positive family history of diabetes. The A1C values in the 4th quartile were higher compared to the 1st and 2nd quartile.

**Table 2:** Characteristics of women with gestational diabetes divided by quartiles of HOMA-IR

		**Insulin resistance- HOMA IR index quartiles (N, mean, range for each quartile)**				
**Variables**		**N = 308**	**N = 319**	**N = 316**	**N = 311**	***p*-Values**
		0.97	1.59	2.37	4.90	
		0.34-1.29	1.29-1.92	1.92-2.89	2.89-20.39	
**Age (years)**	M	29.77	29.83	30.17	30.07	0.7378
	SD	5.36	4.88	5.28	5.05	
**Family history of diabetes**	N	49	66	74	103	<0.0001
	%	17.01	22.68	24.92	36.27	
**Parity**	M	1.98	2.00	1.34	2.01	0.9886
	SD	1.16	1.36	0.07	1.34	
**Gestational age at diagnosis (week)**	M	28.28	28.48	28.78	28.04	0.1752
	SD	4.30	4.49	3.51	4.68	
**BMI (kg/m**^**2**^**)**	M	21.61	22.43	24.75	27.39	<0.0001^1vs3^
	SD	3.25	0.22	4.42	5.12	<0.0001^1vs4^
						<0.0001^2vs3^
						<0.0001^2vs4^
						<0.0001^3vs4^
**Weight gain during gestation (kg)**	M	7.75	8.42	9.12	8.67	0.0058^1vs3^
	SD	4.42	4.64	5.10	6.03	
**Insulin (pmol/l)**	M	33.3	52.2	73.5	134.6	<0.0001^1vs2^
	SD	7.98	2.88	12.78	74.58	<0.0001^1vs3^
						<0.0001^1vs4^
						<0.0001^2vs3^
						<0.0001^2vs4^
						<0.0001^3vs4^
**HbA**_**1c**_**(%)**	M	5.39	5.41	5.53	5.66	<0.0001^1vs4^
	SD	0.45	0.45	0.45	0.51	<0.0001^2vs4^
**HOMA B index**	M	490.47	391.52	361.85	468.08	0.0400^1vs3^
	SD	838.50	30.91	378.98	579.24	
**Glucose at 0 OGTT (mmol/l)**	M	4,72	4.86	4.96	5.36	0.0013^1vs3^
	SD	0.73	0.65	0.70	0.86	<0.0001^1vs4^
						<0.0001^2vs4^
						<0.0001^3vs4^
**Glucose at 2-h OGTT (mmol/l)**	M	8.77	8.69	8.72	8.78	0.7755
	SD	0.96	1.01	1.12	1.34	
**Minimal mean daily dose of insulin (U per day)**	M	9.75	14.29	15.08	19.09	0.0017^1vs4^
	SD	9.43	16.14	15.79	20.80	
**Maximal mean daily dose of insulin (U per day)**	M	19.93	41.02	49.10	59.41	0.0456^1vs2^
	SD	13.98	35.21	37.42	47.51	0.0011^1vs3^
						<0.0001^1vs4^
**Diet + insulin therapy**	N	43	71	79	126	<0.0001*
	%	13,96	22,26	25,00	40,51	

Data are presented as means + −SD for continuous traits or count (percentage) for categorical data.

p values are based on ANOVA test with post-hoc analysis.

The increased HOMA-IR in the 1st quartile was associated with a decreased HOMA-B in the 3rd quartile (p = 0.04).

The minimum mean daily dose of insulin, expressed as international units (IU) per day required to normalise the glucose concentration, was higher in the 4th quartile of HOMA-IR compared with the 1st quartile in the initial phase of treatment, while the maximum mean daily dose of insulin assessed during gestation was significantly higher in the 2nd, 3rd and 4th quartiles in comparison with the 1st quartile.

In the logistic regression analysis, the need for insulin therapy was most consistently associated with insulin resistance index-HOMA-IR value (OR 3.37, 95% Cl 2.10, 5.40), and less consistently with fasting glucose (OR 2.61, 95% CL 1.48, 4.50), age (OR 1.54 95% CL 1.12, 2.11), BMI (OR 1.45 95% CL 1.03, 2.03 ) and weight gain at the diagnosis of GDM (OR 0.72 95% CL 0.52, 0.99). There was no association between insulin therapy and family history of diabetes. Results of these analysis are reported in Table 3.

**Table 3:** Rating logistic regression model parameters for the dependent variable- therapy (diet vs diet + insulin) and independent variables: age, pre-pregnancy BMI, fasting glucose, family history of diabetes, HOMA-IR and weight accretion during pregnancy

**Variables**	**Chi**^**2**^	**β**	**ORs**	**95% Cl**	***p*-Value**
Age	p < 0.0001	0.4302	1.54	(1.12–2.11)	**0.008**
BMI		0.3702	1.45	(1.03–2.03)	**0.033**
Glucose		0.9601	2.61	(1.48–4.50)	**0.001**
Family history of diabetes		0.2078	1.52	(0.82–2.81)	0.187
HOMA-IR		0.4047	3.37	(2.10–5.40)	**<0.0001**
Weight gain at diagnosis of GDM		−0.3287	0.72	(0.52–0.99)	**0.042**

BMI-body mass index, HOMA-IR - insulin resistance index.

Based on logistic regression models, the minimum mean daily dose needed for the initial normalisation of glucose levels was associated with the HOMA-IR index (OR 1.4 95% CL 1.0, 2.0) and with a positive family history of diabetes (OR 1.9, 95% CL 1.1, 3.3) after adjustment for age and BMI. Adjustment for glycaemia in a 2-h OGTT, gestational week as well as weight accretion at diagnosis of GDM the association between the minimum dose and the HOMA-IR does not change. The association is no longer statistically significant after adjustment for fasting glycaemia. The results are presented in Table 4.

**Table 4:** Logistic regression models of the dependent variable minimal mean daily dose of insulin at the beginning of therapy, giving the individual odds ratios (OR) and its confidence interval (CI) for statistically significant parameters

**Variable**	**Chi**^**2**^**(p)**	**β**	**ORs**	**95% Cl**	***p*-Values**
**Model 1**	0.0339				
Age		0.1245	1.1	0.6–2.1	0.6923
BMI		−0.0024	1.0	0.3–3.2	0.9968
HOMA-IR		0.4021	1.5	1.1–2.2	**0.0500**
**Model 2**	0.0155				
Age		0.1347	1.1	0.6–2.1	0.6655
BMI		−0.0643	0.9	0.5–1.9	0.8579
HOMA-IR		0.4474	1.6	1.1–2.2	**0.0100**
Gestational age (week)		0.5278	1.7	0.9–3.3	0.1129
**Model 3**	0.0393				
Age		0.1366	1.1	0.6–2.1	0.6671
BMI		−0.0624	0.9	0.5–1.9	0.8622
HOMA-IR		0.4452	1.6	1.1–2.2	0.0105
Gestational age (week)		0.4804	1.6	0.8–3.1	0.1515
Weight accretion (kg)		0.0098	1.0	0.5–1.9	0.9750
**Model 4**	0.0088				
Age		0.0831	1.1	0.6–2.0	0.7975
BMI		−0.0400	1.0	0.5–2.0	0.9150
HOMA-IR		0.3595	1.4	1.0–2.0	**0.0425**
Family history of diabetes		0.6474	1.9	1.1–3.3	**0.0218**
**Model 5**	< 0.0001				
Age		−0.4710	0.6	0.3–1.3	0.2269
HOMA-IR		0.1989	1.2	0.8–1.8	0.2938
Family		0.1864	1.2	0.6–2.5	0.6115
history of diabetes					
Glucose 0		2.9081	18.3	4.7–70.9	**<0.0001**
**Model 6**	< 0.0001				
Age		−0.5454	0.6	0.3–1.2	0.1627
HOMA-IR		0.4368	1.5	1.1–2.2	**0.0180**
Family history of diabetes		0.3744	1.4	0.7–2.8	0.2635
Glucose 2 h		1.4621	4.3	2.0–9.3	**0.0002**

HOMA-IR- insulin resistance index.

Based on logistic regression models, the maximum mean daily dose of insulin in the course of treatment was associated with HOMA-IR(OR 1.9 95% CL 1.2, 3.0) after adjustment for age, prepregnancy BMI, family history of diabetes and fasting glucose. Further adjustment for 2-h OGTT glucose or weight accretion at diagnosis of GDM does not change this result. Results of this analysis are presented in Table 5.

**Table 5:** Logistic regression models of the dependent variable maximal mean daily doses of insulin during gestation, giving the individual odds ratio (OR) and its confidence interval (CI) for statistically significant parameters

**Variable**	**Chi**^**2**^	**β**	**ORs**	**95% Cl**	***p*-Values**
**Model 1**	0.0009				
Age		−0.1330	0.9	0.4–2.2	0.7699
BMI		0.1357	1.1	0.4–2.9	0.7764
HOMA-IR		0.6324	1.9	1.2–3.0	**0.0105**
Family history of diabetes		0.6093	1.8	0.8–4.2	0.1480
Glucose 0		1.0436	2.8	0.6–12.4	0.1644
**Model 2**	0.0019				
Age		−0.1166	0.9	0.4–2.2	0.7984
BMI		0.1674	1.2	0.5–3.1	0.7299
HOMA-IR		0.6286	1.9	1.2–3.0	**0.0110**
Family history of diabetes		0.6371	1.9	0.8–4.4	0.1347
Glucose 0 Weight accretion (kg)		1.0265	2.8	0.6–12.2	0.1711
		0.1881	1.2	0.5–3.0	0.6794
**Model 3**	0.0008				
Age		−0.3630	0.7	0.3–1.8	0.4546
BMI		0.0454	1.0	0.4–2.8	0.9276
HOMA-IR		0.7091	2.0	1.2–3.4	**0.0070**
Family history of diabetes		0.6468	1.9	0.8–4.5	0.1374
Glucose 0		0.5349	1.7	0.4–8.2	0.5007
Glucose 2 h		1.0370	2.8	1.0–7.8	**0.0447**

HOMA-IR- insulin resistance index.

## Discussion

The first important observation resulting from our study is that significant differences occur in the studied population of women in terms of the severity of insulin resistance evaluated using the HOMA-IR index at the time of GDM diagnosis. According to the International Diabetes Federation criteria, the HOMA-IR cut-off point to differentiate between low and high insulin resistance is 2.38, and HOMA-IR values range from 0.34 to 1.29 in the lowest quartile, whereas in the 4th quartile these values range from 2.89 to 20.39. Such differences in the HOMA-IR indicate a significant pathophysiological variation in the severity of insulin resistance at the diagnosis of GDM in the studied population.

Consistent with our results, several previous studies performed on smaller populations have demonstrated that HOMA-IR index assessed at diagnosis of GDM ranged from 1.6 to 25 [13-17].

Interestingly 1/4 of the women in our study (the first quartile), exhibited a low degree of insulin resistance, while chronic insulin resistance is present in most of the GDM women [18-20], and it progresses in the course of pregnancy.

It is also worth of noticing that the highest rate of insulin resistance (fourth quartile) occurred in 1/4 of our female patients, while most women with this type of diabetes exhibit a mild degree of insulin resistance [18,19].

The results of our study clearly indicate a pathophysiological and clinical variation of GDM in respect to HOMA-IR values. The value of HOMA-IR index higher than >1.29 is associated with a higher value of the pre-pregnancy body mass index, fasting glucose level, insulin level, and severity of GDM. Values of HOMA-IR in the range of 1.92-2.89 are specifically associated with reduced insulin secretion, while values of >2.89 are additionally associated with a higher HbA1c level and an unchanged beta cell function, suggesting insufficient compensation for metabolic demands. It should be emphasised that there were no statistically significant differences between the HOMA-IR quartiles in terms of age, parity and time of diagnosis of GDM.

Our results are consistent with the general opinion that chronic insulin resistance lies at the core of the development of the most common form of beta cell dysfunction present in GDM [21]. The function of the beta cells decreases around a surprisingly low value of insulin resistance – as low as >1.29. The obtained results stress the need for implementing a treatment aimed at improving tissue susceptibility to insulin in GDM women with a HOMA-IR of >1.29. It is especially important to introduce healthy lifestyle changes, as well as a proper diet based on individualized caloric needs and weight gain [22]. It should be pointed out, that limiting excessive growth of fatty tissue may also decrease the adverse effect of adipokines on the insulin secretion [23] and reduce the intensity of insulin resistance of skeletal muscles [6].

Since metformin reduces insulin resistance, metformin treatment seems to be pathophysiologically sound in most of the GDM women, with the exception of subjects with a suspected of developing type 1 diabetes or with type 1 diabetes exhibiting itself in the course of pregnancy. Women with HOMA-IR values in the range of 1.92-2.89 exhibit a decreased beta cell function, suggesting insulin substitution, rather than the introduction of oral hypoglycemic drugs.

It is possible, based on existing evidence that starting insulin substitution in the latter subpopulation of women may delay the development of subsequent disturbances in carbohydrate metabolism [24-26] and thus may partly reduce future cardiovascular risk related to diabetes.

Women with HOMA-IR values of >2.89 are likely to benefit from metformin treatment combined with insulin therapy.

Another interesting finding in this study is the association between the degree of insulin resistance, beta cell function and the severity of GDM. Severity of GDM evaluated by starting insulin therapy and daily insulin dose, exhibits the strongest correlation with HOMA-IR index values. An increase in the HOMA index value of >2.89 is accompanied both by an increase in insulin secretion and by an increase in insulin requirement, which suggests inefficient secretion in response to insulin resistance.

Our findings are in discordance with several up to day published data performed in the small populations of Caucasian GDM women [13,16,17] as well as in Japanese women [27]. In these studies minimal increase in insulin secretion assessed by HOMA B in the range of 80–130 to 170–180 [13,16] or by OGTT- derived indices of beta cell function [27] fail to compensate for increased insulin resistance assessed by HOMA-IR index between 1–1.8 to 2,3 contributed to the development of gestational diabetes. In contrast, we show that basal insulin secretion decrease in the range of HOMA-IR between 1 to 2,39. On the other hand, these results my support our current suggestion that there is a major need for a treatment aimed at improving tissue insulin sensitivity at low insulin resistance index-HOMA-IR values. In another observations, beta-cell activity assessed by median of HOMA-B was equal 154 at the diagnosis of GDM [13] and 200 [17] alongside with the HOMA-IR- 2.1 and 2.4 respectively.

Interestingly, most of these estimates of beta cell activity are lower than those reported in our current study. We suggest that the relatively early week of gestation at the time of diagnosis of GDM as well as a younger age of studied subjects in our data compared with most of other studies [7,13,16,17,28] are partly responsible for these differences. These discrepancies may be due to the small size of populations studied as well as may reflect the differences in diagnostic procedures, severity of glucose intolerance, or anthropometric and ethnical [16] characteristics. In the another study of large size population of GDM women basal insulin secretion assessed by mean of HOMA-B in whole population was equal 200 + −300 while the insulin sensitivity index derived from-OGTT was reduced by 42% versus than in women with normal glucose regulation [28]. These results are in accordance with basal insulin secretory capacity values derived from our current study and suggest pathophysiologic heterogeneity of the studied population of GDM women with respect to this pathophysiological parameter. It is suggested that results from HOMA-B a measure of basal insulin secretory capacity should be interpreted alongside with insulin sensitivity measure by HOMA-IR [29].

In general, our observations seem to be in line with data which investigated insulin resistance during pregnancy and its association with insulin therapy and beta cell dysfunction during pregnancy [30-32].

It has been demonstrated in several of the presented studies that insulin use during pregnancy is both a parameter of GDM severity and a strong predictor for the development of type 2 diabetes [31-33]. Our present results suggest that the degree of insulin resistance, as assessed by the HOMA-IR index at the diagnosis of GDM, could be a potential predictor of GDM severity and of the future development of type 2 diabetes, because this parameter is associated with both beta cell dysfunction and insulin therapy.

The importance of insulin resistance for beta cell function in post-GDM has been partly illustrated by the results of the Troglitazone In Prevention Of Diabetes and Pioglitazone in Prevention of Diabetes studies, in which the protection of beta cell function was closely related to the degree of reduction in endogenous insulin requirements [34].

We acknowledge, that estimation of insulin sensitivity using HOMA-index is less precise than measurement with an insulin clamp, but we consider that HOMA method is more feasible in a large size cohort. Further, we have established, that HOMA would be an accurate measure of total insulin sensitivity thoroughout pregnancy in women with gestational diabetes [35]. Additionally, results of some studies rise the pathophysiologic significance of rather chronic hepatic insulin resistance [36] than the whole-body insulin resistance in declining of beta cell function in GDM women during pregnancy as well as postpartum [37].

A limitation of our study is the use the fasting state HOMA of beta cell function in population of GDM women. There are no evidences that insulin secretion as assessed by HOMA-B index a surrogate of basal insulin secretion is an adequate or is not adequate measure of beta cell function in GDM women. Taken into consideration the results of last data which suggest the pathophysiologic importance of hepatic insulin resistance in GDM women, the evaluation of basal insulin secretion may be a valuable metabolic parameter in these group of women [36-38].

Certain limitation is the lack of information on the dietary habits and the lifestyle prior to the study. Last data revealed, that dietary factors, both before and during pregnancy as well as physical activity are associated with GDM risk [7].

## Conclusions

We conclude that the degree of insulin resistance at the diagnosis of GDM is a marker of GDM severity and pathophysiological heterogeneity.

HOMA-IR values of ≥1.29 at diagnosis may indicate insulin resistance to be the key pathophysiologic mechanism in GDM in the studied population of women. These HOMA-IR values justify aggressive treatment of insulin resistance as a method adequate to the pathophysiologic background of GDM in this population of Caucasian women. Decreased beta-cell function in HOMA-IR values between 1.92-2.89 is associated with decreased insulin secretion. Introduction of insulin treatment in combination with dietary control seems to be more appropriate in treating this subpopulation of women than the administration of oral drugs. HOMA-IR value of >2.89 suggests an inefficient compensation, thus indicating the need for metformin treatment combined with insulin therapy.

Further studies are needed to resolve the problem of optimal treatment strategies, which still remain controversial in this specific, high risk group of women.

## Abbreviations

GDM: Gestational diabetes mellitus; HOMA B: Homeostasis model assessment of beta cell function; HOMA IR: Homeostasis model assessment of insulin resistance; OGTT: Oral glucose tolerance test; BMI: Body mass index.

## Competing interests

The authors declare that they have no competing interests.

## Authors’ contributions

AS conceived the study, design, analysed and interpreted the data and wrote the manuscript, drafted and revised the manuscript. BR contributed to the design of the study, participated in data collection and revised manuscript. KG participated in the sequence alignment and analysis of the data. MW revised and contributed to study design. DR interpreted the data and critically revised the manuscript. All of the authors approved the final version of the manuscript submitted for publication.

## Authors’ information

AS is an academic teacher, a research worker and a clinician at the Nicolaus Copernicus University, head of Diabetologic and Obstetric Care Unit at the University Hospital in Bydgoszcz especially interested in pathophysiology of gestational diabetes, it’s therapy and early prevention of atherosclerosis postpartum.

## Pre-publication history

The pre-publication history for this paper can be accessed here:

http://www.biomedcentral.com/1472-6823/13/21/prepub
